# Is there value in using physician billing claims along with other administrative health care data to document the burden of adolescent injury? An exploratory investigation with comparison to self-reports in Ontario, Canada

**DOI:** 10.1186/1472-6963-5-15

**Published:** 2005-02-18

**Authors:** Beth K Potter, Douglas Manuel, Kathy N Speechley, Iris A Gutmanis, M Karen Campbell, John J Koval

**Affiliations:** 1 Department of Epidemiology and Biostatistics, University of Western Ontario, London, Canada; 2 Department of Epidemiology and Community Medicine, University of Ottawa, Ottawa, Canada; 3 Institute for Clinical Evaluative Sciences, Toronto, Canada; 4 Department of Public Health Sciences, University of Toronto, Toronto, Canada; 5 Children's Health Research Institute, London, Canada; 6 Department of Paediatrics, University of Western Ontario, London, Canada; 7 Southwest Region Health Information Partnership, London, Canada; 8 Department of Obstetrics and Gynecology, University of Western Ontario, London, Canada

## Abstract

**Background:**

Administrative health care databases may be particularly useful for injury surveillance, given that they are population-based, readily available, and relatively complete. Surveillance based on administrative data, though, is often restricted to injuries that result in hospitalization. Adding physician billing data to administrative data-based surveillance efforts may improve comprehensiveness, but the feasibility of such an approach has rarely been examined. It is also not clear how injury surveillance information obtained using administrative health care databases compares with that obtained using self-report surveys. This study explored the value of using physician billing data along with hospitalization data for the surveillance of adolescent injuries in Ontario, Canada. We aimed i) to document the burden of adolescent injury using administrative health care data, focusing on the relative contribution of physician billing information; and ii) to explore data quality issues by directly comparing adolescent injuries identified in administrative and self-report data.

**Methods:**

The sample included adolescents aged 12 to 19 years who participated in the 1996–1997 cross-sectional Ontario Health Survey, and whose survey responses were linked to administrative health care datasets (N = 2067). Descriptive analysis was used to document the burden of injuries as a proportion of all physician care by gender and location of care, and to examine the distribution of both administratively-defined and self-reported activity-limiting injuries according to demographic characteristics. Administratively-defined and self-reported injuries were also directly compared at the individual level.

**Results:**

Approximately 10% of physician care for the sample was identified as injury-related. While 18.8% of adolescents had self-reported injury in the previous year, 25.0% had documented administratively-defined injury. The distribution of injuries according to demographic characteristics was similar across data sources, but congruence was low at the individual level. Possible reasons for discrepancies between the data sources included recall errors in the survey data and errors in the physician billing data algorithm.

**Conclusion:**

If further validated, physician billing data could be used along with hospital inpatient data to make an important and unique contribution to adolescent injury surveillance. The limitations inherent in different datasets highlight the need to continue rely on multiple information sources for complete injury surveillance information.

## Background

The contribution of surveillance systems in providing valuable information for injury prevention and control is widely recognized; for example, surveillance data can be used to highlight the burden of injury, set priorities for prevention, and evaluate preventive strategies [1,2]. Estimates of the population burden of injuries differ, though, depending on how information is obtained. Detailed trauma registries and special surveillance systems [e.g., [3]] contain rich contextual information on particular subsets of injuries, but since such databases are generally not population-based, they cannot be used to estimate the incidence of injury. Although population-based surveys can yield estimates of the total burden of non-fatal injuries across a broad spectrum of injury severity, they often include insufficient sample sizes for studying small population subgroups [4], and are subject to recall errors [5].

Administrative health care databases, due to their presumed near complete coverage of injuries requiring medical care and their lack of reliance on self-reports, may be particularly useful for injury surveillance. Such databases allow for local or regional estimates of the burden of injury, which has been identified as an important goal [2,6,7], and since they are pre-existing, they are cost-efficient. Administrative data also provide an opportunity to examine health care use for injury. Administrative databases only capture injuries that receive medical care, however, and since surveillance using administrative data is often based on hospitalization data alone, only relatively severe injuries are included. Decisions regarding whether to seek medical care and where to seek care for an injury may be influenced by outside factors (such as access to care, care-seeking, and practice patterns), which may lead to selection biases [8-10].

In Ontario, Canada, administrative health care databases that may provide information on the incidence of non-fatal injuries include hospital discharge and physician billing data. Although hospital discharge data have been widely used in Canada to study injury, the feasibility of using physician billing information for injury surveillance has rarely been investigated [11]. These data, if valid, may help to expand the coverage of administrative databases to include more minor injuries, capturing care delivered in physicians' offices and emergency departments. Although minor injuries have less impact on individuals and are less costly to the health care system on a per-injury basis, minor injuries have a large impact in terms of total population morbidity due to their frequent occurrence [12,13]. Expanding coverage by including physician billing data would thus serve to provide a more comprehensive picture of the total health care burden of injury, and may also reduce selection biases in the surveillance data.

It is not clear how the injury information provided by administrative databases compares with that obtained from population-based surveys. A study of adolescent injuries we conducted using data from the Ontario Health Survey (OHS) [14] presented a unique opportunity to explore such a comparison; a subset of the 1996–1997 OHS data was linked by respondent to Ontario administrative health care databases, including both hospital discharge and physician billing data.

The overall purpose of this study was to explore the feasibility and value of using physician billing data for Ontario, Canada, along with hospitalization data, for the surveillance of adolescent injuries. The first objective was to document the burden of adolescent injury based on administrative health care data, focusing on the relative contribution of physician billing information and comparing overall estimates with surveillance information from survey data. The second objective was to examine data quality issues, by directly comparing adolescent injuries identified using administrative health care databases ("administratively-defined injuries") with those identified using self-report survey data ("self-reported injuries").

## Methods

### Sample and data sources

The study sample included adolescents aged 12 to 19 years who participated in the health component of the 1996–1997 OHS (N = 3331), which was part of the National Population Health Survey (NPHS) [14]. Survey responses were linked to administrative health care datasets through unique health card number, respondent name, address, sex, and birthdate. Although over 95 percent of OHS respondents agreed to allow their survey responses to be linked to administrative databases, sufficient information for linkage was available for only 66 percent, including 2067 (62%) of the adolescent participants, due to missing demographic information for respondents. This subgroup provided an opportunity to examine injury occurrence using multiple data sources within the same sample. A unique set of sampling weights was created for the linked subsample of the OHS, to improve representativeness. The linked sample of adolescents was similar to the full OHS sample in terms of gender, rural/urban status, and age. Self-reported information was collected through telephone interviews. Proxy respondents provided survey information for 35 of the 2067 participants.

Inpatient hospitalizations for injuries were identified using the Discharge Abstract Database (DAD) of the Canadian Institute for Health Information (CIHI) [15]. All Ontario hospitals are included in the computerized DAD, which contains clinical, demographic, and administrative data for each hospital discharge. Physician care for injuries, and specifically injuries cared for in emergency departments and in physicians' offices or other outpatient facilities, was identified using physician billing data. Approximately 94% of physicians in Ontario are paid on a fee-for-service basis, through billings to the Ontario Health Insurance Plan (OHIP) [16]. The computerized OHIP claims database captures basic information on these services (Ontario Ministry of Health and Long-Term Care).

### Injury measures

#### 1. Self-reported injuries (survey data)

OHS respondents were asked a series of questions related to acute injuries in the past 12 months that were, from the perspective of the respondent, serious enough to limit normal activities (examples given by the interviewer included "...a broken bone, a bad cut or burn, a sprain, or a poisoning") [14]. Participants reporting that they had experienced one or more such injuries were considered to have a self-reported injury.

#### 2. Administratively-defined injuries

##### i) Hospital visits for injury (identified using hospital discharge data)

Inpatient hospitalizations for injury were identified for the 365-day period prior to the OHS interview, for each adolescent. An adolescent was considered to have an injury-related hospitalization if, during the one-year period, he or she had at least one documented hospital discharge with an External Cause of Injury Code (E Code) in the range 800–999 (excluding codes 870–879 and 930–949, related to medical/surgical misadventures and adverse effects of the therapeutic use of medications), based on the International Classification of Disease (ICD), 9^th ^revision [17].

##### ii) Physician care for injuries (identified using physician billing data)

The OHIP physician claims database does not contain codes representing causes of injury. Rather, injuries were identified based on codes that reflect billable services ("procedure codes"), and the diagnoses associated with such services ("diagnostic codes"). To improve sensitivity, a combination of both diagnostic and procedure codes from the database was used to create an injury algorithm that would identify physician care for injury during the one-year study period, based on the methods of Tamblyn and colleagues [11]. The development of the algorithm was based on a pilot study involving 200 adolescents (further details regarding the algorithm and pilot study findings are available from the authors upon request). Two lists of codes were initially created from the full listing of diagnostic and procedure codes used in the database [18,19]. The first list ("definite injuries") included diagnostic and procedure codes that were viewed as being definitely related to acute injury for adolescents. Since some diagnostic and procedure codes used in the claims data were non-specific, a second list ("possible injuries") was also developed. The initial code lists were reviewed by three physicians with experience in family medicine and/or emergency care. The lists were then expanded and further reviewed by three researchers, including a primary care physician and a researcher with physiotherapy experience. All physician claims with diagnostic or procedure codes on the definite injury list were considered to represent injuries. Based on the pilot test results, claims representing possible injuries were considered injury-related only if they represented care for an adolescent who also had a definite injury claim within a two-day period, and if the possible claim could be considered to represent care for the same injury as the definite claim. The physician billing database also provided information on the location of physician care (e.g., physician's office or emergency department) for each claim.

##### Summary of administratively-defined injury measures

Adolescents who had at least one documented injury in either the hospitalization or the physician billing database were considered to have an administratively-defined injury. Adolescents with documented injury-related physician care at a physician's office or outpatient facility were considered to have a physicians' office visit for injury. Adolescents with documented injury-related physician care at an emergency department were considered to have an emergency visit for injury.

Two alternative injury outcomes were created to examine the impact of decisions made in developing the physician billing algorithm. These outcomes were based on diagnoses that were relatively common and were captured only as possible injuries in the algorithm, including non-specific conditions of the musculoskeletal system and adverse effects of drugs and medications.

### Data analysis

#### Burden of injury

First, the injury algorithm was used with the full sample of 2067 adolescents, resulting in estimates of adolescent injury-related physician care by gender and location of care. Descriptive analysis was then carried out for adolescents with non-missing data on important OHS variables (N = 2047). In addition to examining differences in the overall observed burden of adolescent injury between administrative and survey data, we also explored whether there were differences within demographic subsets of the sample where the types of injuries experienced or the types of injury care received were likely to differ. Thus, we examined the proportion of adolescents with administratively-defined and self-reported injuries separately by gender, age group, and rural versus urban residence. Since numerous comparisons were possible within the results related to the burden of injury, we chose to report 95% confidence intervals around each proportion, rather than presenting statistical tests. Because the OHS used a complex sampling design to yield a provincially representative sample, weighted proportions were calculated. Variance estimates were adjusted using bootstrap replicate weights to account for clustering within the sample [14].

#### Data quality exploration

Two analyses were conducted to explore data quality. First, as a sensitivity analysis for the physician billing database, we examined the impact of re-classifying two common "possible injury" diagnoses as actual injuries in the physician billing database. These diagnoses included i) non-specific musculoskeletal system diagnoses, and ii) adverse reactions to drugs and/or medications. These diagnoses were viewed as potentially problematic because they were not specific enough to injuries to warrant inclusion as "definite" injuries, but we believed that they might be commonly used by physicians providing injury-related care.

Secondly, we directly compared self-reported and administratively-defined injuries at an individual level, to provide further insight into data quality. Not all self-reported injuries identified using the survey data would be expected to have led to medical treatment, and conversely, it is possible that some medically treated injuries may not have led to a restriction in normal activity. Some overlap between the survey data and administrative data was expected, though, in terms of injuries identified. Thus, we examined discrepancies between the injury variables at the level of the individual adolescent (i.e., the extent to which adolescents with administratively-defined injuries were likely to also have self-reported an injury during the same time period). Odds ratios, based on two-way data tables, were used as a measure of association for these direct comparisons of injury variables across data sources. We also explored possible reasons for the discrepancies observed, including potential recall error in the survey data, and potential error in both datasets resulting from overlap between acute injuries and repetitive strain injuries. These exploratory analyses involved, where appropriate, descriptive statistics (e.g., mean or median values) or odds ratios (as a measure of association for two dichotomous variables). All of the data quality analyses were used to examine within-sample methodologic issues. Therefore, as we did not wish to generalize the results from these analyses to a target population, unweighted analyses were conducted, and confidence intervals were not included.

## Results

### The burden of adolescent injury

During the one-year period prior to the OHS interview, there were a total of 13501 physician visits for any cause among the 2067 adolescents in the initial sample (where a visit represents all of the care provided by a physician to a patient on the same date, at any location), based on the physician billing data. Of these, 1390 visits were identified as being related to injury, representing 10.3% of all physician care. The proportion of visits due to injury varied across locations of care. For example, 8.6% of physicians' office visits (n = 604) and 45.6% of emergency department visits (n = 402) were identified as injury-related. A greater proportion of physician care was injury-related among males (14.2%), relative to females (7.3%).

Within the final sample (N = 2047), while 18.8% of adolescents self-reported an activity-limiting injury in the past year, 25.0% had at least one administratively-defined injury, based on the hospitalization and physician billing databases (Table 1, weighted proportions). While 17.1% had at least one physicians' office visit for injury, 13.4% had one or more emergency department visits or inpatient stays for injury (these two outcomes were combined because only 18 adolescents had documented inpatient care for injury). Among adolescents with physician care for injury, the majority had one or two identified visits in the one-year period.

**Table 1:** Sample characteristics and prevalence of injuries by data source (weighted)

	Total (Unwtd N = 2047)		Males (Unwtd N = 1081)		Females (Unwtd N = 966)	
	%	95% CI	%	95% CI	%	95% CI
Rural	17.2	(15.7, 18.7)	17.2	(15.1, 19.2)	17.2	(14.9, 19.6)
Age group						
12–14 years	34.0	(31.5, 36.6)	35.0	(31.6, 38.4)	33.0	(29.2, 36.8)
15–17 years	39.4	(36.7, 42.0)	38.9	(35.3, 42.5)	39.9	(36.0, 43.8)
18–19 years	26.6	(24.1, 29.1)	26.1	(22.8, 29.3)	27.2	(23.3, 31.1)

Injury measures						
Self-reported^1^	18.8	(16.9, 20.7)	22.1	(19.1, 25.0)	15.3	(12.7, 17.8)
Administratively-def^2^	25.0	(22.9, 27.1)	28.0	(24.9, 31.0)	21.7	(18.6, 24.7)
Physician's office^3^	17.1	(15.2, 18.9)	19.5	(16.8, 22.1)	14.4	(11.9, 17.0)
ED/inpatient^4^	13.4	(11.8, 15.0)	15.5	(13.1, 18.0)	11.0	(8.9, 13.1)
1–2 injury visits^5^	16.5	(14.8, 18.3)	18.2	(15.7, 20.8)	14.6	(12.2, 17.0)
> = 3 injury visits^5^	8.2	(6.7, 9.6)	9.7	(7.6, 11.9)	6.4	(4.5, 8.3)

CI = Confidence Interval; def=defined; ED = emergency department; unwtd = unweighted

^1 ^Self-reported injury, identified using survey data

^2 ^Administratively-defined injury, identified in the hospitalization or physician billing databases

^3 ^At least 1 documented physician's office visit for injury within 1 year prior to the interview

^4 ^Any documented emergency department or inpatient visits for injury within 1 year prior to interview

^5 ^Number of physician visits (any location) for injury (based on physician billing data only)

Table 2 shows the proportion of adolescents with each injury outcome, separately for each age group and by rural/urban status. A higher proportion of males were injured relative to females, across injury outcomes and subgroups. An exception was emergency department or inpatient attended injuries among rural adolescents, although in this case, the estimated proportion for females had high sampling variability. A small decrease in the proportion injured was observed with increasing age. This decrease was less apparent for emergency department and inpatient injury visits; again, sampling variability was high. For self-reported injuries, a higher proportion of rural adolescents was injured relative to urban adolescents, particularly for females. Rural/urban differences were not apparent for administratively-defined injuries overall. By location of care, the proportion with a physician's office visit for injury was higher for urban adolescents, while the proportion with emergency department or inpatient injury care was higher for rural adolescents.

**Table 2:** Distribution of injury outcomes (weighted)

Self-reported injury (survey data)						
	Total (Unwtd N = 2047)		Males (Unwtd N = 1081)		Females (Unwtd N = 966)	
	%^1^	95% CI	%^1^	95% CI	%^1^	95% CI

Age 12–14 years	21.2	(17.6, 24.8)	23.4	(18.0, 28.8)	18.6	(13.7, 23.5)
Age 15–17 years	18.9	(15.8, 22.1)	23.7	(19.0, 28.4)	13.9	(9.7, 18.0)
Age 18–19 years	15.6	(12.0, 19.2)	17.8	(12.5, 23.1)	*13.3	(8.2, 18.3)
Rural	21.9	(17.5, 26.3)	23.1	(17.0, 29.2)	20.5	(14.0, 26.9)
Urban	18.2	(16.0, 20.4)	21.8	(18.4, 25.2)	14.2	(11.3, 17.1)

Administratively-defined injury (hospitalization and physician billing data)						
	Total (Unwtd N = 2047)		Males (Unwtd N = 1081)		Females (Unwtd N = 966)	
	%^1^	95% CI	%^1^	95% CI	%^1^	95% CI

Any injury^2^						
Age 12–14 years	27.2	(23.1, 31.2)	29.5	(24.2, 34.9)	24.4	(18.5, 30.4)
Age 15–17 years	25.1	(21.6, 28.6)	27.4	(22.4, 32.5)	22.6	(17.9, 27.3)
Age 18–19 years	22.0	(18.1, 25.8)	26.7	(20.6, 32.7)	17.0	(12.0, 21.9)
Rural	25.2	(20.5, 29.9)	26.6	(20.2, 33.0)	23.7	(17.1, 30.3)
Urban	24.9	(22.5, 27.3)	28.3	(24.7, 31.8)	21.2	(17.9, 24.6)

Phys. office injury^3^						
Age 12–14 years	19.8	(16.2, 23.3)	21.6	(16.7, 26.5)	17.6	(12.5, 22.8)
Age 15–17 years	16.9	(13.9, 19.9)	19.8	(15.2, 24.3)	13.8	(10.0, 17.6)
Age 18–19 years	13.9	(10.5, 17.3)	16.2	(11.1, 21.2)	*11.5	(6.8, 16.3)
Rural	14.0	(10.5, 17.5)	*16.2	(10.8, 21.6)	*11.6	(6.9, 16.3)
Urban	17.7	(15.6, 19.8)	20.1	(17.1, 23.2)	15.0	(12.0, 18.1)

ED/inpatient Injury^4^						
Age 12–14 years	13.8	(10.8, 16.9)	15.9	(11.5, 20.4)	*11.4	(7.4, 15.4)
Age 15–17 years	13.6	(11.1, 16.2)	14.5	(10.9, 18.2)	12.7	(8.9, 16.4)
Age 18–19 years	12.4	(9.4, 15.4)	16.4	(11.7, 21.1)	*8.2	(4.7, 11.7)
Rural	18.0	(13.9, 22.1)	17.6	(12.5, 22.8)	*18.4	(12.1, 24.6)
Urban	12.4	(10.6, 14.2)	15.1	(12.3, 17.9)	9.5	(7.3, 11.7)

CI = Confidence Interval; ED = emergency department; phys. = physician's; unwtd = unweighted

^1 ^Row percentages (for example, of 12–14 year-olds, the percent who experienced the injury outcome)

^2 ^Any documented injury in the administrative databases (hospitalization and physician billing data)

^3 ^At least 1 documented physician's office visit for injury within 1 year prior to the interview

^4 ^Any documented emergency department or inpatient visits for injury within 1 year prior to the interview

* Proportion should be interpreted with caution due to high sampling variability

### Sensitivity analysis and comparison of injuries across data sources

As a sensitivity analysis for the physician billing data algorithm, the impact of re-classifying two common "possible injury" diagnoses as actual injuries was examined. When the non-specific musculoskeletal system diagnoses were added to the injury dataset, the proportion of adolescents with administratively-defined injury increased from 25.0% to 29.3%. When adverse reactions to drugs and/or medications were added, the proportion increased only slightly, to 25.9% (weighted proportions).

The results of the within-sample analysis used to compare injuries identified using different data sources are shown in Table 3 (unweighted). Section i) of the table shows the direct comparison of administratively-defined and self-reported injuries at the individual level, for the total sample, and then separately by gender, and by location of care for administratively-defined injuries. For example, of the 2047 adolescents in the final sample, 550 had a documented administratively-defined injury, while 1497 had no such injury. Of the 550 adolescents with administratively-defined injury, 213 (38.7%) self-reported an injury, compared with 193 (12.9%) among the 1497 adolescents with no administratively-defined injury. The odds ratio for the relationship between administratively-defined and self-reported injury was 4.3. There was a higher congruence between the two data sources in terms of identified injuries for females (odds ratio 5.7), relative to males (odds ratio 3.4). To examine the congruence with self-reported injury separately by location of care for the administratively-defined injuries, the third and fourth columns of the table ("administratively-defined injury") were restricted to those identified as having specifically received care at either a physician's office or an emergency department/inpatient facility. For example, 358 adolescents had a documented physician's office visit for injury, and 134 (37.4%) of these adolescents also self-reported an injury. When compared with the 12.9% of adolescents who self-reported an injury but had no administratively-defined injuries, the resulting odds ratio was 4.0. There was a higher congruence with self-reported injuries for emergency department or inpatient care for injury (odds ratio 5.8).

**Table 3:** Exploring self-reported versus administratively-defined injury (unweighted)

i) Direct comparison of injuries identified using different data sources: self-reported injury for those with and without administratively-defined injury							
		Administratively- defined injury			No administratively- defined injury		
	Total N	N	Self-report N(%)		N	Self-report N(%)	Odds ratio

Total	2047	550	213 (38.7)		1497	193 (12.9)	4.3

Males	1081	318	126 (39.6)		763	123 (16.1)	3.4
Females	966	232	87 (37.5)		734	70 (9.5)	5.7

Phys. office^1^	1855	358	134 (37.4)		1497	193 (12.9)	4.0
ED/ inpatient^2^	1812	315	145 (46.0)		1497	193 (12.9)	5.8

ii) Time from most recent administratively-defined injury to OHS interview (N = 550)^3^							

					N	Mean (# days)	Median (# days)

Adolescents with self-reported injury					213	141	125
Adolesents without self-reported injury					337	173	171
Total					550	160	146

iii) Self-reported repetitive strain injuries, by self-reported & administratively-defined acute injuries (N = 2045)							

					N	Repetitive Strain Injury N (%)	Odds Ratio

Self-reported acute injury					406	57 (14.0)	1.9
No self-reported acute injury					1639	129 (7.9)	

Administratively-defined acute injury					550	88 (16.0)	2.7
No administratively-defined acute injury					1495	98 (6.6)	

Administratively-def. & self-rep. acute injury					213	35 (16.4)	1.1
Administratively-def. & no self-rep. acute injury					337	53 (15.7)	

def = defined; ED = emergency department; OHS = Ontario Health Survey; phys = physician's; self-rep. = self-reported

^1 ^At least 1 documented physician's office visit for injury within 1 year prior to interview, based on the administrative data. Adolescents with emergency department or inpatient visits but no physicians' office visits for injury are excluded from the denominator.

^2 ^Any documented emergency department or inpatient visits for injury within 1 year prior to interview, based on the administrative data. Adolescents with physicians' office visits but no emergency department or inpatient visits for injury are excluded from the denominator.

^3 ^Analysis includes only those adolescents (N = 550) with administratively-defined injury

In order to investigate the possibility that recall error may have led to underreporting in the survey, we investigated the relationship to recall time (Table 3, section ii). For adolescents with at least one administratively-defined injury, those who self-reported an injury had a shorter recall time from the most recent documented administratively-defined injury to the OHS interview (median 125 days) compared with adolescents who did not self-report an injury (median 171 days).

Finally, to explore whether some acute injuries may have been misreported as repetitive strain injuries in the OHS (based on a series of questions on repetitive strain injuries that preceded those on acute injuries), and to explore whether the algorithm used with the physician billing database may have led to misclassification of some repetitive strain injuries as acute injuries, we examined the relationship to self-reported repetitive strain injuries. Both self-reported acute injury and administratively-defined acute injury appeared to be related to self-reported repetitive strain injury (odds ratios 1.9 and 2.7 respectively, Table 3, section iii), although the relationship for administratively-defined injury was stronger. Among those with administratively-defined injury, there was no strong evidence of a relationship between self-reports of acute injury and repetitive strain injury; repetitive strain injury was reported by 16.4% of the 213 adolescents with self-reported acute injury, and a similar 15.7% of the 337 adolescents without self-reported acute injury (Table 3, section iii, last two rows).

## Discussion

### Contribution of physician billing data to injury surveillance using administrative databases

This exploratory study focused on the potential value of using physician billing data in combination with hospital discharge data to document the burden of injuries among adolescents. The results suggest that adding physician billing claims to hospitalization information is a feasible method of improving the comprehensiveness of healthcare administrative datasets. Approximately 10 percent of all physician care for adolescents in the study was identified as injury-related. Although a smaller proportion of physicians' office visits was identified as injury-related, relative to emergency department physician visits, office care actually represented a larger number of visits. Thus, these relatively more minor injuries represent a large component of adolescent injury morbidity that would be missed if estimates relied on hospital data alone or even on a combination of hospital and emergency care information. The observed differences in the rural/urban distribution of adolescent injuries by location of care (Table 2), reflecting potential difference in injury severity or access to care between rural and urban adolescents, also highlight the importance of capturing information across the full spectrum of care.

### Comparison of administrative databases and self-reports: value for injury surveillance

A higher proportion of adolescents was identified as having administratively-defined injury relative to self-reported injury. One might expect the definition of self-reported injury used in the survey (injuries that limit normal activities) to capture a broader spectrum of injury severity compared with the administrative data (since some activity-limiting injuries may not receive medical care). The higher proportion of adolescents with administratively-defined injury, though, suggests that there may also be a subset of medically treated injuries that do not in fact limit normal activities; in other words, perhaps the definition of injuries used in the survey was actually more restrictive. Although neither data source can be viewed as a "gold standard", these results suggest that administrative health care data may actually provide a more sensitive means of ascertaining injuries, relative to self-reported survey data. Injuries identified as medically treated using administrative data may also be viewed as representing the health concerns of the person seeking care, and they have an impact on the health care system.

These findings highlight the potential importance of administrative databases as a source of population-based injury information that can be used for affordable ongoing surveillance and for examining health care system issues such as patterns of service delivery. Despite these advantages, a limitation of many claims datasets, including the OHIP database, is a lack of detail on the circumstances surrounding the occurrence of injuries. The billing data contained no external cause information, such that description of injuries by mechanism and intent was not possible.

### Exploring data quality issues: injury outcomes in administrative databases and self-reports

Although the distribution of injuries, particularly for gender and age, was fairly similar for self-reported and administratively-defined injuries (Table 2), congruence of injury outcomes was relatively low at the individual level (Table 3, section i). This may in part reflect the different definitions of injury represented in the datasets (medically treated injuries in the administrative data, versus activity-limiting injuries in the survey data). Our exploration of data quality issues, however, revealed potential errors in both databases that may have contributed to the discrepancies.

In the survey data, we found some evidence of recall errors (Table 3, section ii). This finding is supported by previous research documenting recall errors in self-reports for a variety of health outcomes, including chronic conditions [e.g., [20,21]] as well as injuries [e.g., [5,22,23]]. For example, in a study of parental recall of non-fatal injuries in children and adolescents, estimates of annual injury rates were found to decline as the recall period for injuries increased from two weeks to 12 months [5]; this suggests that the 12 month recall period used in the 1996–1997 OHS may have led to underreporting. In the study of parental recall referred to above, more severe injuries (resulting in surgery or hospitalization; or resulting in restriction to bed or school absence) appeared to be less subject to recall errors, relative to minor injuries [5]. This may partly explain the stronger association we found between self-reported injuries and administratively-defined injuries when administratively-defined injuries were restricted to those identified as having received emergency department or inpatient care. Studies that have directly compared self-reported health care use with health care use identified in medical records across a variety of health services have also tended to find that both males and females underreport physician visits to a greater extent than hospital or emergency care, particularly as the recall period increases [e.g., [24-26]].

In addition to recall error in the survey data, inaccuracies in the administrative databases may have played a role in contributing to the discrepancies between the administrative and survey data. Errors in the DAD have been identified [27], although this likely had little impact, due to the small number of injury hospitalizations. The method used to identify injuries in the physician billing database was exploratory. The sensitivity analysis and results related to repetitive strain injury (Table 3, section iii) highlight the need to further validate the physician billing data algorithm, ideally using comparisons with medical charts. With respect to repetitive strain injury, its stronger observed relationship with administratively-defined injury (as compared with self-reported acute injury) suggests that the physician billing data algorithm may have led to the inclusion of some repetitive strain injuries. The lack of evidence for a relationship between self-reported acute injury and repetitive strain injury among those with administratively-defined injury (Table 3, section iii) suggests that confusion with repetitive strain injury in the survey did not lead to underreporting of acute injuries.

### Strengths and limitations

Strengths of our study included the detailed exploration of the methods used to identify injuries using physician billing data, and the unique comparison of injuries across datasets within the same sample of adolescents. In addition to the need to further validate the algorithm used with the physician billing data, study limitations included the small sample size, particularly for investigating the distribution of injuries resulting in emergency and inpatient care, and the incomplete linkage of the survey and administrative datasets. Although the incomplete data linkage may reduce the generalizability of the study findings, the linked sub-sample was similar to the full Ontario survey sample across demographic characteristics (gender, rural/urban status, and age), and the unique sampling weights created for this sub-sample may have improved representativeness. Finally, because this study capitalized on an opportunity presented by a larger study on youth injuries, we focused specifically on adolescents. Further research could examine the generalizability of both the approach and the findings to other age groups, where the types of injuries experienced and care-seeking patterns may differ. Studies in jurisdictions with similar medical claims databases would also help in assessing the generalizability of our research.

## Conclusion

Collectively, our findings allow us to draw two main conclusions. First, the results suggest that there is potential value in using physician billing data along with other administrative health care databases for the surveillance of injuries among adolescents. Although they are lacking in details about the circumstances surrounding injuries, comprehensive administrative injury datasets may be particularly useful for describing the overall occurrence of injury at local or regional levels, and for describing the economic implications of injury for the health care system. Secondly, we identified data quality concerns in both the survey and administrative databases that suggest a need for improvement and further study; for example, further research could help to identify appropriate recall periods and question wording for minimizing errors in survey data, and to determine the level of detail needed to accurately identify injuries in administrative databases. Because various sources of data are susceptible to different limitations, it remains important to consult multiple sources of information to fully document the burden of injury [2,4,7].

## Competing interests

The author(s) declare that they have no competing interests.

## Authors' contributions

BKP participated in the design and coordination of the study and the development of the physician billing algorithm, carried out all statistical analyses, and drafted the manuscript. DM participated in the design, coordination, and supervision of the study and development of the physician billing algorithm, as well as revisions to the manuscript. KNS participated in the design, coordination, and supervision of the study, as well as revisions to the manuscript. IAG participated in the design of the study and development of the physician billing algorithm, as well as revisions to the manuscript. MKC participated in the design of the study and revisions to the manuscript. JJK participated in the design of the study and revisions to the manuscript, and provided statistical guidance. All authors read and approved the final manuscript.

## Pre-publication history

The pre-publication history for this paper can be accessed here:


